# Immunomodulatory Effect of Electromagnetic Field in the Treatment of Traumatic Brain Injury

**DOI:** 10.26502/jbb.2642-91280069

**Published:** 2023-02-01

**Authors:** Tye Patchana, Devendra K Agrawal, David Connett, David Baron, Dan Miulli

**Affiliations:** 1 Riverside University Health System, Department of Neurological Surgery, USA; 2 College of Osteopathic Medicine of the Pacific, Western University of the Health Sciences, Pomona, California, USA; 3 Arrowhead Regional Medical Center, Department of Neurological Surgery, USA

**Keywords:** Electromagnetic Field, Immunomodulation, Traumatic Brain Injury (TBI), Neuroinflammation, Excitotoxicity

## Abstract

A review was performed utilizing PubMed and GoogleScholar to highlight the future directions of EMF research in the setting of brain pathology, specifically in ischemic and traumatic brain injury. Additionally, a critical review of the current state-of-the art of EMF use in treating brain pathology has been conducted. The authors have added to this large body of research their own experimental studies, including a description of the on-going studies. The field of EMF utilization in the diagnosis and treatment of brain injury is highly promising and warrant careful studies in clinically relevant experimental models followed by human trials in TBI.

## Introduction

Medicine has a history of several thousand years. During this time, patients have been continuously treated people with drugs and surgery. Surgery has been and is currently performed with a knife, usually cutting away tissue, rarely adding material. Lesions can be grossly removed but not on a cellular level, and therefore many cancers remain incurable. Chemotherapy affects the whole body, and many times with adverse effects. Conventional methods are limited in targeting a specific organ, tissue, or cell. However, recent advancements in science allowed the development of novel techniques and tools to treat some diseases with lasers, ultrasonic dissectors, and even focused beam radiation. The next era of medicine will begin when we can target specific sites in a pathological lesion using the new technology. However, it is critical to understand the underlying cellular, molecular, genetic, and epigenetic mechanisms of the disease process for precision and accuracy, and then control the direction of the treatment. However, this may not change the immediate chemistry of the cell and the tissue. It is critical to understand life on a molecular layer or smaller, and then use this information to control the organelles, the cells, the tissues, the organs, and the entire body, both in physiological and pathological conditions. It is now well established that electrons spin, protons vibrate, and molecules vibrate. Harnessing this vibration information may allow us to control almost all aspects of medicine. This is not an unusual concept. Indeed, ultrasound and radiation, with the help of electrical energy are used in the diagnosis, treatment, control of a disease process, and possibly cure. We even depend upon the natural vibrational signal of hydrogen when we use an MRI scanner. There has already been precedence exploring the resonance of targets and influencing those targets with the same vibration. Just as two opposite vibrational sound waves make silence together, a specific frequency may silence a deadly killer or bring back the sweet sound of life. Electrical energy using Pulsed Electrical Magnet Field (PEMF) is used in spinal cord stimulators to relieve pain, in bone stimulators to heal non unions, in tremors to calm the hands, and in the heart to capture a beat. Indeed, such techniques require a multidisciplinary approach with the integration of expertise and tools in chemistry, physics, biology, anatomy, physiology, pathology, and clinical medicine. The purpose of this review is to critically analyze the concept and the scientific foundation underlying PEMF-induced immunomodulation of traumatic injury in the brain. Furthermore, a discussion is provided on potential modification of the currently designed and tested equipment for experimental models to understand the underlying cellular and molecular mechanisms and examine the effect of PEMF in the management of traumatic brain injury.

## Methods

A comprehensive literature search utilizing PubMed and Google Scholar was conducted between January 2001 through December 2022. Articles published in English were reviewed and included in this article. The following key words were utilized: EMF stroke, electromagnetic field, cerebrovascular accident, pulsed electro-magnetic field (PEMF), EMF, and traumatic brain injury, brain trauma, neuroprotection and EMF, immunomodulation, and various iterations. The findings were critically reviewed, and the information was synthesized into this comprehensive review article highlighting the clinical application and future directions.

## Types and characteristics of traumatic brain injury

The inclusion of the myriad ways in which traumatic brain injury occurs under one umbrella term “Traumatic Brain Injury (TBI)” continues to make it difficult to identify therapeutic targets. TBI occurs in the setting of Motor Vehicle Accidents (MVA), falls, blast injuries, and penetrating brain trauma. The primary selection criteria for inclusion into TBI trials have been the GCS scoring system [1]. According to the current paradigm in TBI, there is an initial precipitating event (primary injury) followed by a clinicopathological cascade of events resulting in secondary injury [2]. Epidemiological studies have identified that mild, moderate, and severe TBI is a widespread pathology, and the milder forms of TBI may escape inclusion in the database [3]. Recent studies investigating the dynamics of TBI and MVA have demonstrated that the majority of TBIs involve skull fracture, Subarachnoid Hemorrhage (SAH), focal injury, and Subdural Hematoma (SDH) with the moderate-to-severe pathology in majority of the cases [4]. Penetrating brain injuries are a considerable type of TBI in both civilian and military personnel with mortality rates that can approach 90% in some studies [5]. Management of the patients who survive the primary injury hinges on the attenuation of the secondary injury resulting from cascading cellular events. Blast type injuries are also prominent in law and military personnel, leading to cognitive deficit, attention, and sleep impairments. Interesting, it was recently found that repeated low-to- moderate intensity blasts demonstrate similar neuropathological effects as one large blast [6].

## Pathophysiology of Traumatic Brain Injury

**Figure 1:**
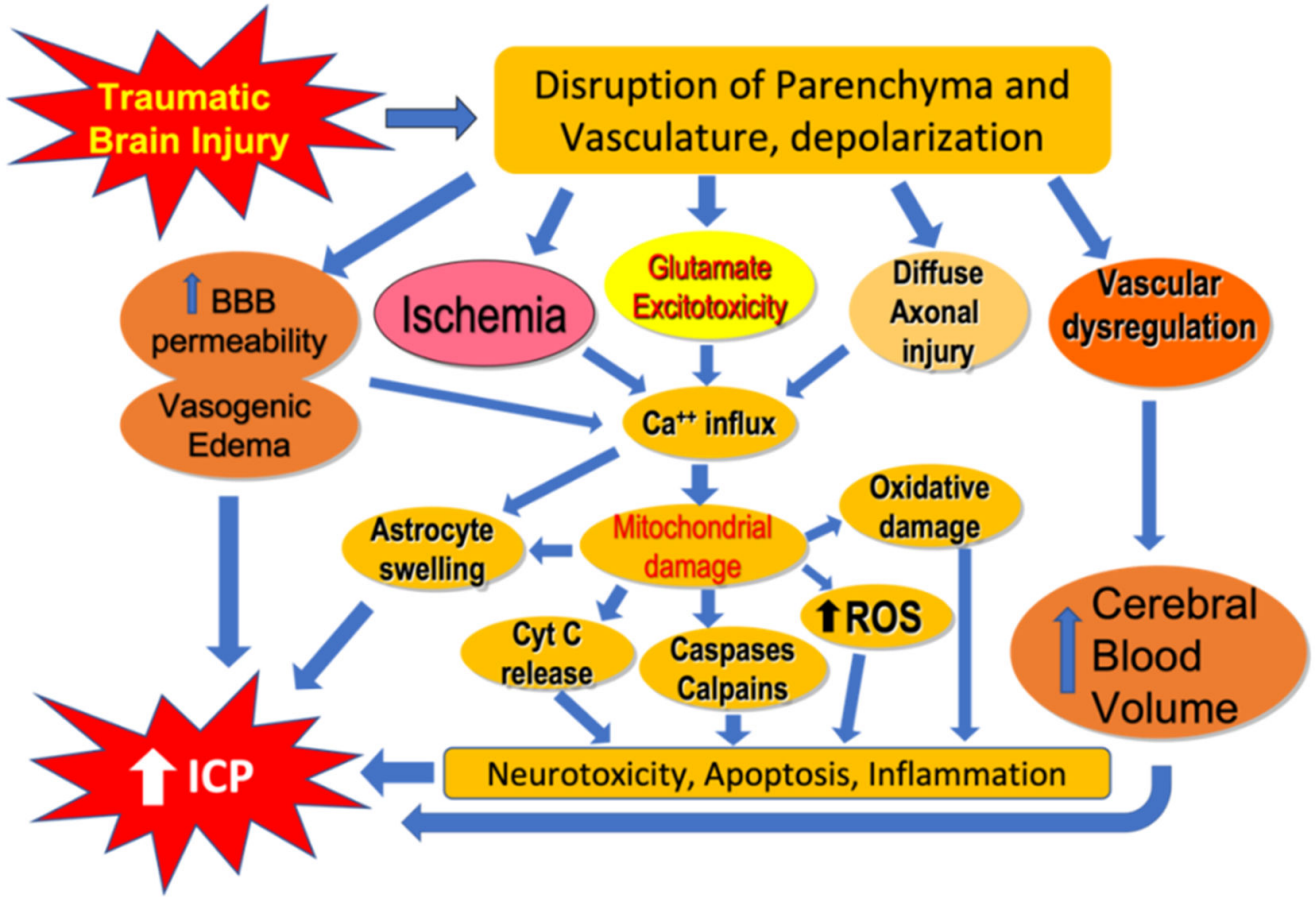
Schematic diagram showing the pathophysiology of traumatic brain injury resulting in increased Blood Brain Barrier (BBB) permeability, vasogenic edema, diffuse axonal injury, ischemia, mitochondrial dysfunction damage and increased Reactive Oxygen Species (ROS), neurotoxicity, apoptosis, inflammation in the brain. Vascular dysregulation results in increased cerebral blood volume and cerebral swelling. All these cellular events result in Increased Intracranial Pressure (ICP) and neurological deficits following traumatic brain injury.

Seminal to the definition of traumatic brain injury are temporary or permanent dysfunction or dysregulation of neural tissue resulting from external forces. These impairments can manifest as an altered state of consciousness, physical, psychological, and cognitive impairments [7]. As discussed, a primary inciting event occurs in TBI, followed by a series of events in which clinicians may intervene to improve outcomes. This secondary injury includes excitotoxicity, disruption of mitochondrial function, and accumulation of oxidative stress, lipid peroxidation, inciting apoptosis, and neuroinflammation (Figure 1).

Neuroinflammatory changes following TBI have similarities to brain injury following ischemic or reperfusion events [8]. Prostaglandins, cytokines (interleukins, chemokines, growth factors, etc.) that are pro-inflammatory, Reactive Oxygen Species (ROS) including free radicals comprise mediators are activated [9-10]. The overall result of these mediators is a disruption of the normal permeability of the Blood Brain Barrier (BBB), which in turns causes edema and resultant neurological deficits (Figure 1). This neuroinflammation may even be prolonged over the course of years, as has been shown by Bye et al. with activation of macrophages, that in turn activate microglial cells, which increase release of astrocytes [11]. Failure of the normal homeostatic mechanisms of the BBB cause the release of excess neurotransmitters that have been implicated in TBI. Of these, glutamate is notable as failure of glutamate transporters has been implicated in the TBI cascade of events [12-13]. With release, glutamate and its associated metabolites bind to glutamate receptors causing activation and inducing excitotoxicity [8]. Among these, N-methyl-D-aspartate (NMDA) and Alpha-Amino-3-Hydroxy-5-Methyl-4-Isoxazoleproprionic Acid Receptor (AMPA) receptors allow influx of sodium, potassium, and calcium with resultant depolarization [14]. Excess calcium is implicated in the apoptotic cascade-including calpain, calcineurin, and caspases [15]. Recent evidence has correlated calpain to GCS in patients with TBI [16]. Mitochondrial dysregulation damage and oxidative stress are also implicated in the TBI cascade. Indeed, the influx of calcium itself into mitochondria may result in Reactive Oxygen Species (ROS), causing membrane depolarization that hinders ATP synthesis and further dysregulates the system [17]. Lastly, at a brain neuronal circuit level, shear stress on the longitudinally oriented neural structures leads to Diffuse Axonal Injury (DAI) (Figure 1). The resultant shear stress causes destruction to the axonal cytoskeleton, microtubules and neurofilaments [18].

## Current Treatment and management strategies in TBI

The current paradigm in TBI management and treatment hinges on protection of the neural elements from the secondary injury discussed above. Initial goals remain protection of the airway and the circulatory system (Table 1). Given that hypotension doubles mortality, and hypoxia and hypotension triples mortality, support of these systems is paramount [19]. The Brain Trauma Foundation guidelines further recommend Intracranial Pressure (ICP) monitoring in patients with GCS3-8 with abnormal CT of the head or a normal CT scan with age greater than 40, motor posturing, or systolic blood pressure less than 90mmHg (http://braintrauma.org/coma/guidelines). At our institution, following hemodynamic stabilization and ICP control, we aim for initiation of early nutrition, physical occupational speech therapies, and if necessary, tracheostomy and percutaneous gastronomy tube placement. Additionally, our institution incorporates osteopathic manipulative treatment, which has been shown to decrease ICP [20] (Table 1). In Marino, et al [21], the authors review the connection between cerebral concussion, brain edema, and other diseases and how the glymphatic’s neurophysiology changes with different conditions. The glymphatic flow and drainage can be enhanced with treatments that affect the peri-arterial CSF influx route, the perivenous efflux route, and the transparenchymal route. These are currently facilitated with medications, procedures, and osteopathic manipulative treatment and may be further enhanced by manipulating the brain neuronal circuit with EMF.

## Overview on Different Types of EMF

**Figure 2:**
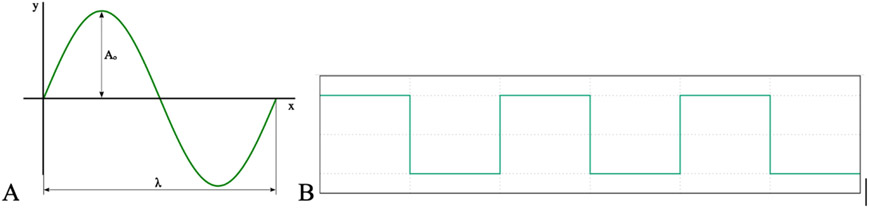
Different types of EMF used for stroke treatment, including (A) EMF based on sinusoidal waveform and frequency, and (B) Pulsed waveform, indicative of PEMF.

In addition to following the Brain Trauma Foundation Guidelines for the Management of Severe Head Injury, which depends on best medical and surgical care, TBI may be further diagnosed and treated using a new technology based on the brain’s electromagnetic field. 92% of patients with severe head injury suffer five minutes of ischemia and infarction is found at autopsy in 80% of patients dying of severe head injury [22-25]. Clearly, ischemia and infarction greatly affect patient outcomes in TBI and can be modulated with recognition and treatment. Current treatment includes, as stated above best medical and surgical care. Measuring and modulating the brain EMF may change outcomes as well. Figure 2 demonstrates the different types of EMF that have been used in stroke treatment as outlined by Moya Gomez et al., 2021 [26]. Several types of EMF may be utilized. Stationary Magnetic Field (SMF) requires an electromagnetic field with the same direction and magnitude due to a suppressed electrical component or 0 Hz frequency [26]. Low energy, time varying magnetic fields typify Pulsed Electro-Magnetic Field (PEMF). These low energy electric fields are comprised of specific shapes and amplitudes (Markov 2007) and may be found in a variety of different patterns including asymmetric, biphasic, quasi-rectangular or triangular [27]. Sinusoidal electromagnetic fields are non-pulsed (SEMF), in contrast to the on and off nature of PEMF, follows a sinusoidal waveform [26]. Non-pulsed SEMF has been shown to have a modulation effect on reactive oxygen species, on nitrous oxide species (NOS), and have neuroprotective effects in ischemia of the rat model [28].

## Electromagnetic Field and Radio Frequency in Ischemic Stroke

Low energy, time varying magnetic fields are Pulsed Electro-Magnetic Field (PEMF). Several studies have shown beneficial effects of PEMF on ischemic models [29-32]. The fields can be generated under various conditions from the purely electrical, to the magnetic, to the Radio Frequency (RF). This is athermal energy used since 1970s in the healing of bony non-unions. Each time that there is an electrical field there is a magnetic field. A varying electrical field does cause a varying magnetic field. Two methods for activating cells in the motor cortex utilizing PEMF, have been developed. This technique does not require direct contact with the surface of the brain. Merton and Morton introduced the first method, Transcranial Electrical Stimulation (TES), in 1980. It utilizes a high-voltage electrical pulse (up to 2 kV) of a short duration (less than 10 us) delivered through electrodes placed on the scalp with the anode over the cortical region to be stimulated (anode stimulation). This produces a magnetic field that passes largely unimpeded through the tissues of the head, in contrast to electric fields, which are diffused by head tissue. Oscillations in the magnetic field, in turn, induce an electrical current in the brain. The strength of the induced current is, in part, a function of the rate of change of the magnetic field, which varies with the current in the coil. Barker and CO-workers first reported the other form of noninvasive cortical cell activation, Transcranial Magnetic Stimulation (TMS), in 1985. They used a high-current (4 kA) and short-duration (110 us) pulse width. This was discharged through a coil placed on the scalp over the motor strip to produce a brief magnetic field, resulting in secondary currents inside the brain, which then depolarized cells within the motor cortex. For a given magnetic pulse, the direction of current flow through the coil, as well as the shape, size, and windings of the coil, play important roles in determining the effectiveness of TMS [33]. Both TMS and TES have limitations in depth of penetration. RF does not have these draw backs and in fact can and does penetrate all tissue levels. The distance traveled is proportional to the frequency.

Like the electrical stimulation of the exposed motor cortex in cats, monkeys and humans, spinal cord recordings during human spinal surgery have confirmed that single transcranial stimuli, either electrical or magnetic, evoke multiple descending volleys in humans. At above-threshold intensities, the shortest latency direct wave results from depolarization of the neuronal circuit of the pyramidal cell axons. Subsequently occurring indirect waves likely originate from nearby cortical neurons, which then excite pyramidal cells via synaptic connections. Due to their transynaptic origin, the number and amplitude of indirect waves are influenced by the excitability level of the motor cortex. Multiple descending volleys travel through the fast-conducting portion of the corticospinal tract and summate at the segmental motor centers to produce firing of alpha-motor neurons, which ultimately results in fairly synchronized short-latency Motor Evoked Potential (MEPs). This is what occurs daily as the neurophysiologists monitors patients during surgery. The current human stimulation of specific neuronal circuits during surgery is based upon years of research detailing the neuronal circuit and brain electrical activity. Individual cellular electrical action potentials have also been studied using an invasive probe. The action potential occurs over 1 millisecond (ms) (1000Hz); it is hyperpolarized for 2-3 ms (300 Hz) at +15 mV with a relative refractory period of 15 ms, yielding a relative Action Potential (AP) generation rate of 66.6 Hz. Post-synaptic potentials arrive at a frequency of 1000 Hz. Cell maintenance occurs at 30-5000 Hz. Additionally, inserting clinical micro-recording devices the frequencies of groups of cells have been discovered. Nordhausen developed a 3D silicon contact electrode array which records 100 separate channels of neuronal activity. Rousche and Normann reported that this same type of contact array called the Utah Intracortical Electrode Array stimulates and records in a single layer up to 1.5 mm beneath the surface. Cochlear cells have a frequency of 1000-4000 Hz at −20 to −80 mV, subthalamic nuclei 37 Hz, globus pallidal external cells 40 Hz, substantial nucleus reticulata 75 Hz, and globus pallidal interna cells 80-90 Hz. Individual atoms are known to vibrate at individual Larmor frequencies. The frequencies at 1 Tesla are H+ 42.6 MHz, H^2^ 6.5 MHz, P^31^ 17.2 MHz, C^13^ 10.7 MHz, N^14^ 3.1 MHz, N^15^ 4.3 MHz, S^33^ 3.3 MHz, F^19^ 40.1 MHz. These frequencies are detected and “tuned in” by non-contact magnetic resonance imaging.

There is a wide range of varying electrical fields in the neuronal population. Rappaport [34] developed some of the initial investigations into this field with a PEMF utilizing a 27.1 MHz carrier that was modulated by 400 Hz pulse on rats [34]. The most studied electrical potentials are those obtained from electrodes inserted into the scalp. This is the coordination of brain activity. The activity occurs over a frequency from 1 to 50 Hz and from 2 to 100 μV. Specifically, EEG activity is classified as Beta (14-30 Hz), alpha (8-13 Hz), Theta (4-7 Hz), and delta activity (0.5-4 Hz). EEG activity is the summation of the cortical thickness of 2.5 mm, comprising a surface area of 2300 cm^2^, with a density of 10,000 neurons per mm^3^, yielding approximately 10^10^ neurons. Apart from the EEG, visual evoked potentials have frequencies of 1-100 Hz, brain stem auditory evoked responses range from 100 to 3000 Hz, and cognitive functions (P300) are measured at 3.3 Hz and +4 μV extending to P900 1.1 Hz. Memory generally occurs at 2.5 Hz and 10 μV. In a rabbit model of ischemic stroke, PEMF reduced edema as assessed by MRI and histological studies [35]. These EMF signals have been measured in detail in a continuous non-contact fashion at a distance and as a result, in a similar fashion the EMF signal can be transmitted to affect the brain neuronal circuit. At the cellular level, EMF has been found to have affect a large range of biological properties [36]. These include but are not limited to nitrous oxide modulation [37] superoxide modulation [38], apoptosis modulation [39] modulation of microcirculation [40], and modulation of inflammation and edema [41]. Penaphilippides et al., 2016 demonstrated cytokine/chemokine gene expression modulation by PEMF in mice studies with PEMFs demonstrating a suppression of inflammation in stroke, albeit in the later period [42]. Additionally, apoptosis indicators have been shown to be decreased by PEMF [43]. However, Bates et al. did find no improvement in functional or histological outcomes in a rat model [44]. It is known that both brain trauma and acute ischemic injury have similar mechanisms, involving cascades of cellular events with blood brain barrier disruption and influx of immune cells that further cause damage [45]. From the authors’ perspectives, trauma is stroke and inflammation.

There are limited reports on the investigation of EMF effect in TBI. Nonetheless, PEMF has been shown to have both anti-inflammatory and pro-regenerative effects in both animal models and in human trials. PEMF has been implicated in the reduction of both neuroinflammation and infarct size, highlighting it as a potential post-stroke therapy [42]. Normal mitochondrial function is imperative for the normal production of ATP and associated cellular function. During cerebral ischemia, this process is negatively affected, with hypoxia resulting in a breakdown of the aerobic respiration pathway in mitochondria. This results in lack of ATP and breakdown of the Na+/K+ pump, cellular swelling, and eventual neuronal death. PEMF has been found to act as a modulator of the adenosine receptor. This increases the function of endogenous adenosine with upregulation of A2A and A3 receptors in neuronal cells, with resulting neuroprotection [46]. Though studies have shown modulation of lipid peroxidation in rat models, the overall results are unclear, though suggesting protection to various cell types [47]. In view of the limited reports, there are several outstanding questions that warrant detailed careful investigations. These include: the effect of EMF on mitochondrial function and lipid peroxidation in brain cells; the effect of EMF on neuroinflammation, the release of excitatory amino acids from presynaptic nerve terminals in the brain, infiltration of inflammatory immune cells following injury, release of inflammatory cytokines, axonal degeneration, apoptosis, and impaired autophagy; the effect of EMF on oxidative stress; and EMF induce neuronal regeneration. There is no conclusive data on the effect of EMF in large animals in traumatic brain injuries.

## The future of neuromodulation by EMF

There is a great need for the treatment of severe head injury, ischemic, and hemorrhagic stroke. There may be a revolution in the treatment of neurological disease. We have treated this system with surgery to cut parts out and medicine to try to add a change to the whole system to achieve the desired isolated effect. We expect to influence a cellular process by bathing it with chemicals, but it may be more probable that we can affect the organs, cells, and organelles on their level. We can cause these changes by utilizing the intrinsic properties. Of course, medication will continue to be needed to provide the substrate for reactions to take place. Indeed, the electrical energy is necessary and can be manipulated on all levels by the neurological system. Neuromodulation utilizes and changes the innate system in fighting disease in a continuous non-contact manner from a distance. However, utilizing the current treatments of Transcranial Magnetic Stimulation (TMS) and Transcranial Electrical Stimulation (TES) physicians can stimulate a large area of the intact human brain. The technologies have been applied generally, to affect an outcome, similarly to the discovery of x-rays and its treatment of lice and many other conditions. Radiation treatment has advanced to include focusing numerous sources of gamma radiation into pinpoints, at certain dosages, to kill tumor cells or desensitize nerve pain. The current EMF technology can in the intact human brain, in real-time, continuously, at a distance, identify specific brain neuronal circuits, the specific signals from those circuits, and pinpoint a continuous treatment to that diseased neuronal circuit, with real-time feedback. Such treatments may consist of an externally applied helmet and wave guide. The helmet and wave guide have been constructed. The EMF sensors easily pick up EMF activity in a non-contact manner through the scalp from any part and depth of the brain. The brain neuronal circuit EMF characteristics may then be adjusted. The transmitter and receiver sensors operate from 0.01 Hz to GHz. Voltage sensitivity is from picovolts to millivolts. Pulse width range from nanoseconds to milliseconds. Patterns recognition is completed with artificial intelligence analyzing the EMF wave. The pattern orientation of the transmitted EMF is adjusted to account for the different directions of the signals obtained. The depth of penetration and area of transmission will initially be measured and adjusted in the pig model. Once the appropriate EMF measurement are obtained in the traumatic pig and human, and a modulated PEMF applied in the pig trauma model, a modulating PEMF may be able to be applied in the human traumatic brain injury in the hospital setting, continuously, in a non-contact manner. This new diagnosis and treatment could then be applied at the time of brain injury in the human at the scene until specific injuries can be ascertained and appropriate frequencies applied. These may be frequencies to inhibit some areas while stimulating other areas to survive. This helmet and waveguide can be shaped to allow the insertion of ICP monitors and measure the frequencies of the dysfunctional brain. After stabilization of damage the helmet and waveguides would be applied during rehabilitation.

This same type of modulation can also take place during regular learning. A 3.3 Hz, 10 μV pulse to re-enforce the education would follow a stimulus. Southworth reported on the Alpha Stim CES to enhance attention and the ability to learn new tasks. Other uses would be in psychological conditions. The cap could be applied in the acute setting of trauma and stroke and worn during the chronic phase of trauma and stroke to affect memory, concentration, impulsivity, and other behaviors. Cyberonics promotes the Vagus nerve stimulator in the treatment of treatment resistant major non-psychotic depression. Utilizing data obtained from the shark’s electrical signature, the helmet and waveguides could be modulated to that frequency to inhibit cancer growth. Psychological symptoms such as cognitive deficit, impulsivity, mania, depression, anxiety, and a host of neuropsychiatric conditions are unfortunately common following TBI. Additionally, the pharmacological treatments of these conditions have been studied in few controlled clinical trials [48]. Unfortunately, the chronic sequala of TBI usually does not garner as much attention in the literature as the acute manifestations, though a large number will go on to have persistent sequelae of neuropsychological problems [49]. Studies have shown that a reduction in oxidative stress may occur with low frequency EMF utilized in post-acute stroke rehabilitation patients [50]. In this setting and in trauma, neuromodulation may offer a treatment with fewer side effects than medications. Future studies will help to elucidate this.

## Future studies

One of the primary goals of this article is to suggest possible future modulations of neuronal circuits in complex neurological diseases such as the physiological and psychological ramifications of head trauma and stroke. The project is divided into multiple parts. The first involves measuring the normal frequencies, electrical activity and magnetic field in the pig and human. Animal models will need to be used because the microenvironment is important negating single cell systems. The “signal” will be the electrical and magnetic signature, calibrated by amplitude, duration, type of wave, frequency, and flux. This will involve applying advanced technology as well as modifying the current technology. The information will have to be processed and refined. After the information is obtained in the normal state it will have to be obtained in the diseased state. Next, in the pig TBI model EMF in a continuous non-contact fashion will be applied to potentially reverse neuronal circuit damage. Finally, this type of information can be used to treat human problems of head trauma and stroke including its psychological components. A helmet and waveguide detector will be placed on the pig model to look for reproducible frequencies. Prior studies, as stated above, demonstrate information in the Hz range, which is the normal EEG, and encourage the investigation, quantification, and qualification for EMF signals in the kHz, MHz, THz, and GHz range. The second phase will be the recording after clinical lesioning, reproducing trauma or stroke in the pig model, once again looking for the differences in the information, from normal to abnormal. The lesion will be trauma, and secondary ischemia injury and anoxic injury. The third phase would be the neuromodulation of abnormal brain cell circuits, cellular, and psychological abnormalities: brain infarction injury, ischemia, trauma, and anoxic injury. This device can be applied easily and using telemetry would allow the treatment of patients at a distance. Some of the challenges to bringing PEMF modulation in the setting of brain injury to fruition involve the lack of a standardized setting for EMF use. Within the literature there is a large array of differences in the use of wave type, intensity, type of technology, frequency usage, and exposure time creating confounding variables [41]. Overall, there is convincing data that demonstrate that EMF is effective in the treatment of brain injury, whether that injury occur via acute ischemia or traumatic brain injury, which has been corroborated by animal studies.

In our initial testing, proprietary induction sensors (patented, Model BS-1000, Quasar Federal Systems, San Diego, CA) were used to measure the EMF of specific human brain activities in a non-contact and non-invasive fashion using a prototype lightweight helmet as previously described by Wiginton et al. and Brazdzionis et al. [51-53]. This helmet was constructed using two layers of Mu-metal (MuMETAL^®^, Magnetic Shield Corporation, Bensenville, IL) with inner and outer layers of interlaced copper mesh to absorb and reflect the external EMF signal. Both layers of Mu-metal were separated approximately 2.5 cm. Four holes were drilled within the helmet to accommodate four 18-inch induction sensors. These four sensors (Bx, By, Bz, and B319) were configured to bitemporal and bifrontal locations as previously described [51-53]. These induction sensors require a 9-volt power supply and consume 3.3 mA in order to measure non-direct current, low-magnetic fields in a single axis between 1 Hz and 30 kHz through the use of a solenoid coil around a 0.2 inch diameter high-permeability core. Each sensor was ensured to be positioned with the positive end (+) oriented toward the scalp. Furthermore, the Bz sensor was placed on the right side at approximately the right motor strip with the trajectory pointed toward the left motor strip, the By sensor was placed at the right temporal region with a trajectory oriented toward the left motor strip, the B319 sensor was placed on the left side at approximately the location of the left motor strip with a trajectory orienting toward the left motor strip, and the Bx sensor was placed in the left temporal region with a trajectory pointed toward the left motor strip. Each sensor was placed within plastic Polyvinyl Chloride (PVC) piping. The PVC piping containing the sensor was then wrapped in Mu-metal to form an EMF channel to function similarly to a waveguide to focus the generated EMF by targeted brain tissue and exclude external EMF signal. Sensor recordings were recorded on a laptop and post-processed as described by Wiginton et al. using Fast Fourier Transformation (FFT) [3]. Detection sensitivity for sensors was set at 1.5 pTrms/rtHz at 1 Hz, 0.15 pTrms/rtHz at 10 Hz, 0.025 pTrms/rtHz at 100 Hz, and 0.02 pTrms/rtHz at 1 kHz and above. Sensor responses were captured between 1 Hz and 2 kHz, and a gain filter module was used set with a 10x gain for amplifying signal with a 2-kHz low-pass filter for anti-aliasing. Data capture was completed at 5,000 samples per second. Each test was conducted with a non-ferromagnetic metronome placed outside the Faraday cage containing the human participant, helmet, and sensors. This metronome produced a sound at 120 bpm (2 Hz) to synchronize activities and to detect a reproducible recording of brain activity from each subject. Utilizing a helmet, electromagnetic field channels will be connected to the helmet construct with the sensors placed within each channel. Thin lines connecting the helmet to the amplifier and gain filter module and from the amplifier to the computer and recording equipment represent wires connecting the devices [52]. Volunteer human subjects performed each test by participating in designated reproduced activities. Subjects were placed supine and donned the helmet. Activities involving hand or foot tapping were completed at a rate of 2 Hz. Of note, within the Faraday cage, subjects were asked to keep their eyes closed to limit visual stimulus. Furthermore, the shielded room and Faraday cage were dark to limit visual stimulation. Mechanistically, hand tapping was completed through the protocol delineated by Brazdzionis et al. [52]. This tapping activity entailed lifting the hand as a singular unit while leaving the wrist fixed on the ground. The tips of the fingers were elevated 6 cm and then brought back to the ground. Each elevation and return to baseline consisted of a single tap. Foot tapping was completed with the knees and hips flexed. Each foot was dorsiflexed off the ground with the heel remaining fixed on the ground. Toes were elevated 6 inches through dorsiflexion and plantarflexed back to the ground. Each dorsiflexion and plantar flexion cycle was designated as a single tap. Each test started with 30 seconds of "rest" wherein the participant was asked to not tap and try to maintain a lack of higher cortical thoughts. This rest was followed by trials of 60 seconds of the designated activities with 30-second rest "breaks" between each 60-second activity bin. For analysis, summative FFT transformed data placed into 20-second bins were plotted graphically. Testing was completed to investigate the ability and the effects of brain attenuation of external magnetic fields on stereotyped motor activities. This was done to assess the effectiveness of measurement of the generated magnetic field in the brain and the effects of external magnetic field transmission of the generated field. Further investigation included evaluating sensors from a distance when using Mu-metal shielding as an EMF channel functioning like a waveguide to evaluate the decay of the magnetic field over distance.

## Conclusion

Overall, this review has highlighted some of the future directions of this novel EMF research in the setting of brain pathology, specifically in ischemic and traumatic brain injury. Additionally, a critical review of the current state-of-the art of EMF continuous non-contact treatment of brain injury insults has been conducted. The authors have additionally added to this large body of research their own experimental studies, including a description of the on-going studies. Challenges include completing studies to streamline the process and evaluate therapeutic and diagnostic metrics. However, the novel field of EMF utilization in the diagnosis and treatment of brain injury is highly promising and warrant careful studies in clinically relevant experimental models followed by human trials in TBI.

## Figures and Tables

**Table 1:** Current treatment strategies in traumatic brain injury.

Intervention	Effect	Notes
AIRWAY	Secure airway for oxygenation	Hypoxia and hypotension Triples mortality
CIRCULATION	Supports cerebral perfusion pressure	Hypotension doubles mortality
ICP MONITORING	Monitoring ICP, divert CSF If needed	Maintain cerebral perfusion pressure, prevent ischemia
SUPPORTIVE MEASURES	Early therapy, Rehabilitation, nutrition	Early rehabilitation to improve neural function
